# Combined PI3Kα-mTOR Targeting of Glioma Stem Cells

**DOI:** 10.1038/s41598-020-78788-z

**Published:** 2020-12-14

**Authors:** Frank D. Eckerdt, Jonathan B. Bell, Christopher Gonzalez, Michael S. Oh, Ricardo E. Perez, Candice Mazewski, Mariafausta Fischietti, Stewart Goldman, Ichiro Nakano, Leonidas C. Platanias

**Affiliations:** 1 Robert H. Lurie Comprehensive Cancer Center of Northwestern University, 303 East Superior Street, Lurie 3-220, Chicago, IL 60611 USA; 2 Department of Neurological Surgery, Feinberg School of Medicine, Northwestern University, Chicago, IL USA; 3 Division of Hematology/Oncology, Department of Medicine, Feinberg School of Medicine, Northwestern University, Chicago, IL USA; 4 Division of Hematology/Oncology/Stem Cell Transplantation, Department of Pediatrics, Ann & Robert H. Lurie Children’s Hospital of Chicago, Chicago, IL USA; 5 Department of Neurosurgery and O’Neil Comprehensive Cancer Center, University of Alabama at Birmingham, Birmingham, AL USA; 6 Medicine Service, Jesse Brown VA Medical Center, Chicago, IL USA

**Keywords:** CNS cancer, Cancer stem cells

## Abstract

Glioblastoma (GBM) is the most common and lethal primary intrinsic tumour of the adult brain and evidence indicates disease progression is driven by glioma stem cells (GSCs). Extensive advances in the molecular characterization of GBM allowed classification into proneural, mesenchymal and classical subtypes, and have raised expectations these insights may predict response to targeted therapies. We utilized GBM neurospheres that display GSC characteristics and found activation of the PI3K/AKT pathway in sphere-forming cells. The PI3Kα selective inhibitor alpelisib blocked PI3K/AKT activation and inhibited spheroid growth, suggesting an essential role for the PI3Kα catalytic isoform. p110α expression was highest in the proneural subtype and this was associated with increased phosphorylation of AKT. Further, employing the GBM BioDP, we found co-expression of *PIK3CA* with the neuronal stem/progenitor marker *NES* was associated with poor prognosis in PN GBM patients, indicating a unique role for PI3Kα in PN GSCs. Alpelisib inhibited GSC neurosphere growth and these effects were more pronounced in GSCs of the PN subtype. The antineoplastic effects of alpelisib were substantially enhanced when combined with pharmacologic mTOR inhibition. These findings identify the alpha catalytic PI3K isoform as a unique therapeutic target in proneural GBM and suggest that pharmacological mTOR inhibition may sensitize GSCs to selective PI3Kα inhibition.

## Introduction

Glioblastoma (GBM), classified as WHO grade IV glioma, is the most prevalent and malignant primary brain tumour and is essentially incurable^1^. Currently, maximal surgical resection followed by chemoradiation and adjuvant temozolomide treatment is the standard-of-care, resulting in a median overall survival of 14.6 months^2^. Because of the very poor outcomes and aggressive behaviour of these tumours, considerable efforts have been made to extensively characterize GBM at the molecular level. Initially, four different GBM subtypes were identified through comprehensive patient sample analyses^3,4^. However, single-cell transcriptome analysis revealed the existence of only three GBM subtypes designated as proneural (PN), mesenchymal (MES), and classical (CL) GBM^5^. Additionally, a report using epigenomic profiling allowed the sub-classification of GBM into six categories, based on distinct DNA methylation profiles^6^. Although these efforts have advanced our overall molecular understanding of GBM, they have not yet resulted in effective targeted approaches that might improve clinical outcomes.

GBM tumours are not homogeneous neoplasms but rather represent ecosystems that contain diverse neoplastic populations, including cancer stem cells (CSCs). CSCs are believed to contribute to recurrence in multiple cancer types, but the exact mechanisms underlying such recurrence are unclear^7–9^. Brain CSCs play key roles in GBM progression because of their enormous capacity for proliferation, self-renewal, and multilineage differentiation; characteristics that are important for tumour-initiation in serial transplantation experiments^10,11^. In recent years, numerous studies provided strong evidence for glioma stem cells (GSCs) being associated with intratumoural cellular heterogeneity^4,5,12–16^ and plasticity^17–20^, both major factors contributing to the poor prognosis and recurrence of GBM patients. These characteristics have fuelled the concept that therapeutic approaches must include strategies tailored to target the GSC population to block GBM growth and prevent recurrence.

Increasing evidence indicates that key aspects of CSC function are dependent on phosphatidylinositol-3 kinase (PI3K) signalling^21^, while activation of the PI3K/AKT/mammalian target of rapamycin (mTOR) pathway is associated with poor prognosis in GBM patients^22^. Heterodimeric Class I_A_ PI3Ks are composed of a catalytic subunit (p110α, p110β, or p110$\updelta$, encoded by the genes *PIK3CA*, *PIK3CB*, or *PIK3CD*) and a p85-type regulatory subunit^23^. PI3Ks are antagonized by the tumour suppressor phosphatase and tensin homolog (PTEN)^24^. Hence, alterations in the PI3K and PTEN pathways can be frequently found in different human malignancies, including GBM. For instance, 89.6% of GBM tumours have at least one alteration in the PI3K pathway and 39% have two or more^3^. Specifically, 59.4% of GBM show mutations in PI3K genes or *PTEN* mutations/deletions with the majority affecting p110α and/or p85α subunits^3^. Therefore, alterations in the PI3K and PTEN network resulting in aberrant signalling activity may provide unique therapeutic opportunities for treatment interventions in GBM.

There is evidence indicating that PI3K/AKT signalling stimulates gliomagenesis specifically in brain CSCs because neural progenitors expressing the progenitor/stem cell marker nestin are particularly prone to AKT-driven oncogenic transformation^25^. Also, in the perivascular niche, nestin expressing cells exhibit increased PI3K/AKT activation, indicating a role for PI3K/AKT in neural progenitor cells residing in their stem cell niche^26^. Hence, in mouse medulloblastoma models, PI3K/AKT promotes survival and radioresistance of CSCs in the perivascular niche^27^. Importantly, in GSCs the regulatory PI3K subunit p85 directly interacts with CD133, a marker for both neural stem cells and brain CSCs, and this CD133 association promotes activation of the PI3K/AKT pathway, providing further evidence for a distinct and crucial role for PI3K in GSCs^28^. Given that CD133 expressing cells contribute to glioma radioresistance^17^, inhibition of PI3Kα might be an effective approach for specific targeting of therapy resistant GSCs.

Given the essential contributions of PI3K/AKT for GSCs and GBM development, pharmacological PI3K inhibition might represent a promising strategy for this disease. However, pan-PI3K inhibitors have limited potential as they exhibit only a narrow therapeutic window^29^. Studies investigating distinct roles of PI3K isoforms suggest that isoform-selective PI3K inhibitors might show better target selectivity, resulting in reduced toxicities^23^. Particularly, inhibitors targeting the alpha catalytic PI3K isoform have emerged as potentially effective but less toxic^30^. In fact, PI3Kα specific inhibitors have demonstrated encouraging efficacy in GBM^31^. Alpelisib (BYL719) is such a PI3Kα selective inhibitor that has demonstrated a favourable safety profile and encouraging activity in patients with solid tumours^32^. Additionally, in initial studies, alpelisib has shown antineoplastic effects in GBM and GSCs^33^. However, there is evidence suggesting that selective PI3Kα inhibition requires simultaneous targeting of additional pathways, such as mTOR signalling, to efficiently block compensatory survival signalling^34–36^. Cancer cell growth is largely dependent on lipid metabolic processes, which are tightly controlled by mTOR signalling, a pathway that is commonly perturbed in cancers^37^. In response to targeted intervention, multiple mechanisms can converge on mTOR to rewire these processes, thereby promoting cancer cell survival and therapy resistance^38^. This suggests mTOR represents a key target for combinatorial anti-cancer strategies.

In the present study we explored the roles of PI3Kα and mTOR in GBM and GSCs. Analysis of gene expression data indicated elevated expression of *PIK3CA* in the PN GBM subtype as compared to CL and MES subtypes. Moreover, elevated coexpression of *PIK3CA* and *NES* (the gene encoding nestin) was associated with poor prognosis in this GBM subtype. We employed PN and MES GSC lines and found greatly elevated phosphorylation of AKT in PN GSCs, indicative of increased PI3K activity. Concomitantly, neurosphere growth of PN GSCs was potently inhibited by alpelisib and these effects were significantly enhanced when combined with pharmacological mTOR inhibition. Altogether, we provide evidence for a distinct role for *PIK3CA* in PN GSCs that suggests increased vulnerability of this GBM subtype to PI3Kα targeted combinatorial approaches.

## Results

### Alpelisib inhibits PI3K/AKT signalling and exhibits antineoplastic effects in GBM cells

In initial studies, we used a panel of conventional GBM cell lines to assess the efficacy of alpelisib, a PI3Kα-selective inhibitor that has shown encouraging activity in patients with solid tumours^32^. Alpelisib inhibited PI3Kα signalling in a dose dependent manner, as reflected by the reduced phosphorylation of AKT on Ser-473 in U87 (Supplementary Fig. S1A), LN18 (Supplementary Fig. S1B) and LN443 (Supplementary Fig. S1C) GBM cells. Similarly, cell viability was inhibited in a dose responsive manner in all three lines (Supplementary Fig. S1D–F), as was anchorage independent growth of colonies in soft agar (Supplementary Fig. S1G–I). As alternative mTOR pathways may sustain cancer cell survival in the presence of alpelisib and PI3Kα inhibition^34–36^, we examined whether concurrent mTOR inhibition potentiates alpelisib mediated inhibitory effects. The combination of alpelisib and the catalytic mTOR inhibitor OSI-027 potently blocked phosphorylation of AKT(Ser^473^) in U87 (Supplementary Fig. S2A), LN18 (Supplementary Fig. S2B) and LN443 (Supplementary Fig. S2C) cells, albeit with variable efficacies. Notably, the combination of alpelisib and OSI-027 reduced viability of GBM cells significantly, as compared to treatment with either drug alone (Supplementary Fig. S2D–F). Also, the combination of alpelisib and OSI-027 potently suppressed colony formation as judged by anchorage independent growth in soft agar (Supplementary Fig. S2G–I).

### Pharmacological mTOR inhibition enhances the growth inhibitory effects of alpelisib in nestin expressing GBM spheroids

**Figure 1:**
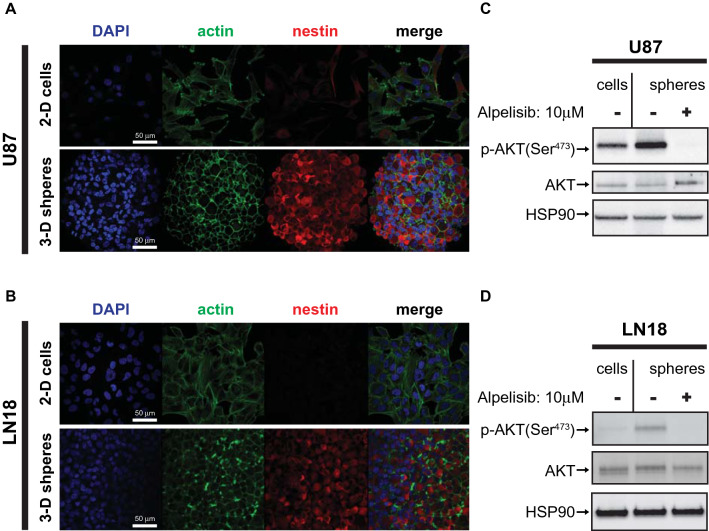
Increased PI3K/AKT activation in nestin expressing 3-D spheroids. (**A**, **B**) U87 (**A**) and LN18 (**B**) cells were grown in 2-D adherent cell culture (top panels) or as 3-D spheroids in cancer stem cell medium (bottom panels) and stained for DNA (blue), actin (green) or nestin (red). Corresponding 2-D and 3-D confocal microscopy images were acquired using identical settings. Scale bar, 50 µm. (**C**, **D**) U87 (**C**) or LN18 (**D**) cells were grown as 2-D monolayer (cells) or 3-D spheroids (spheres), treated with alpelisib (10 µM, 90 min), and subjected to immunoblotting using rabbit anti p-AKT(Ser^473^) or HSP90 or mouse anti AKT antibodies simultaneously, followed by detection using anti-rabbit HRP or anti-mouse AlexaFlour488 secondary antibodies.

**Figure 2:**
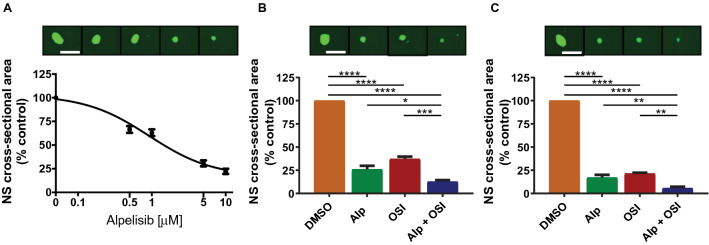
Effects of dual PI3Kα and mTOR inhibition in GBM spheroids. (**A**) U87 GBM cells were grown as spheres in CSC medium for at least 5 days. Spheres were dissociated and seeded at 500 cells/well into round-bottom 96-well plates in the presence of increasing concentrations of alpelisib as indicated. After 7 days, spheres were stained with acridine orange and imaged to determine cross-sectional area. Data represent means ± SEM of 5 independent experiments, each done in triplicate. Representative images are shown in the top panels. (**B**, **C**) Same experiment as in (**A**) using 5 µM alpelisib and 1 µM OSI-027 (**B**) or 10 µM alpelisib and 2 µM OSI-027 (**C**). Data represent means ± SEM of 6 independent experiments, each done in triplicate. Representative images are shown in the top panels. Unpaired one-way ANOVA, *p ≤ 0.05; **p ≤ 0.01; ***p ≤ 0.001; ****p ≤ 0.0001.

There is evidence indicating that in vitro three-dimensional (3-D) spheroid cultures may more accurately reflect the complexity of solid tumours than simple 2-D cell monolayers^39,40^. To study the role of PI3Kα in GBM spheroids, we cultured U87 and LN18 GBM cells under serum-free conditions in 3-D. Both U87 and LN18 cells formed spheres under these culture conditions (Fig. 1). Next we immunostained GBM spheres for the intermediate filament protein nestin whose expression is associated with increased self-renewal capacity and the ability to differentiate into multiple cell types^41,42^. While nestin expression was largely absent in GBM cells grown as 2-D monolayers (Fig. 1A,B, upper panels), cells grown as GBM spheres in 3-D exhibited strongly increased expression of nestin (Fig. 1A,B, lower panels). This suggests that GBM cells adopt CSC characteristics, which is consistent with previous studies that have reported that U87 GBM cells adopt a more “stem-like” phenotype and acquire some self-renewal capacities when grown as GBM spheroids^43,44^. Additionally, we observed greatly increased phosphorylation of AKT (Ser^473^) in GBM spheres as compared to their 2-D counterparts, suggesting activation of the PI3K/AKT pathway in GBM spheroids (Fig. 1C,D). This phosphorylation of AKT on Ser-473 was efficiently blocked by the PI3Kα specific inhibitor alpelisib, indicating this AKT phosphorylation is largely dependent on the PI3K alpha catalytic isoform in this experimental setting (Fig. 1C,D). The increased AKT phosphorylation indicates that neurosphere cultures may require high PI3K/AKT activity for survival and growth. In line with this notion, alpelisib potently blocked growth of U87 spheroids (Fig. 2A) and this effect was even more pronounced when alpelisib was combined with pharmacological mTOR inhibition (Fig. 2B,C). These results suggest that GBM spheroid growth depends on activation of the PI3K/AKT/mTOR pathway, and dual inhibition of PI3Kα and mTOR potently blocks growth of GBM spheres in 3-D.

### Vulnerability to PI3Kα selective inhibition is increased in PN GSCs and additional mTOR targeting enhances these inbhibitory effects in PN and MES GSCs

**Figure 3:**
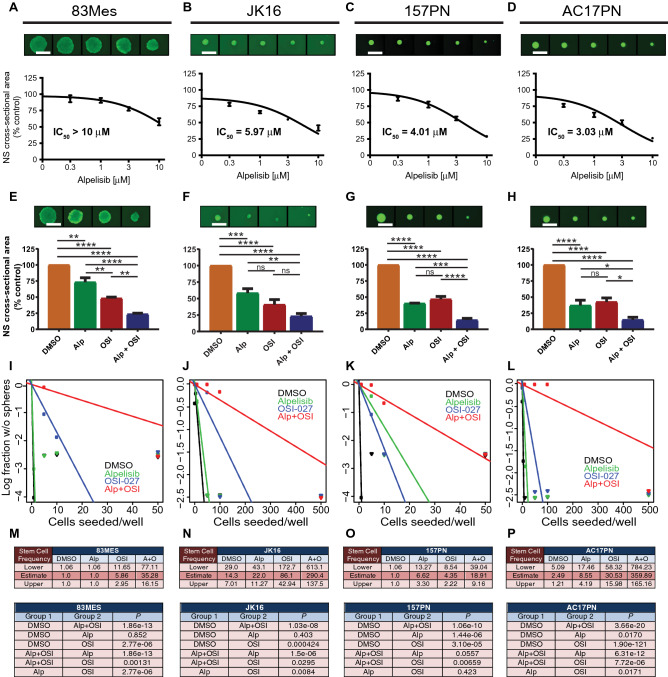
Effects of dual PI3Kα and mTOR inhibition in GSCs. (**A**–**D**) 83Mes (**A**), JK16 (**B**), 157PN (**C**), and AC17PN (**D**) GSC lines were seeded at 500 cells per well into round-bottom 96-well plates in the presence of increasing concentrations of alpelisib as indicated. After 7 days, spheres were stained with acridine orange and imaged on a Cytation3 instrument using Gen5 v 2.09 software to determine cross-sectional area. Prism GraphPad 8 was used to determine IC_50_ values. Data represent means ± SEM of 4 (83Mes, JK16), or 3 (157PN, AC17PN) independent experiments, each done in triplicate. Representative images are shown in the top panels. (**E**–**H**) 83Mes (**E**), JK16 (**F**), 157PN (**G**), and AC17PN (**H**) GSC lines were seeded at 500 cells per well into round-bottom 96-well plates in the presence of alpelisib (5 µM) and/or OSI-027 (1 µM). After 7 days, spheres were stained with acridine orange and imaged to determine cross-sectional area. Data represent means ± SEM of 4 (83Mes, JK16), 3 (157PN), and 5 (AC17PN) independent experiments, each done in triplicate. Representative images are shown in the top panels. Unpaired one-way ANOVA, *p ≤ 0.05; **p ≤ 0.01; ***p ≤ 0.001; ****p ≤ 0.0001. (**I**-**P**) GSCs were subjected to in vitro limiting dilution assays (n = 3, each done in duplicate) plating decreasing number of cells (500; 100; 50; 10; 5; 1 cells per well) in the presence of alpelisib (5 μM) and/or OSI-027 (1 μM). ELDA for 83Mes (**I**,**M**), JK16 (**J**,**N**), 157PN (**K**,**O**), and AC17PN (**L**,**P**) was done using the ELDA software (http://bioinf.wehi.edu.au/software/elda/). (**M**-**P**, upper panels) Stem cell frequencies of GSCs were estimated as the ratio 1/*x* with the upper and lower 95% confidence intervals, where 1 = stem cell and *x* = all cells. (**M**-**P**, lower panels) *P*-values from chi-square analysis of group comparisons.

Accumulating evidence indicates GSCs are key contributors to GBM initiation, progression, recurrence and resistance^11,45^. Hence, curative approaches must include strategies tailored to target GSCs. To study the effects of selective PI3Kα inhibition in GSCs, we employed the GSC neurosphere model system. These neurosphere cultures propagated in serum-free medium under stem cell-permissive conditions produce multipotent neurospheres capable of self-renewal^46,47^ that are enriched for cells competent to initiate tumours^48^. All established patient-derived GSC lines cluster in groups representing the PN or MES subtypes only^15,18^. We used patient-derived GSC lines 83Mes, 157PN, AC17PN^19^ and JK16^49,50^ to determine the effects of alpelisib on GSC lines. Initial experiments determined the dose response effects on GSCs grown as neurospheres. We found that GSC lines of the MES subtype were less responsive with half maximal inhibitory concentration (IC_50_) values of over 10 µM for 83Mes (Fig. 3A) and 5.97 µM for JK16 (Fig. 3B), whereas PN GSC lines were more sensitive with IC_50_ values of 4.01 µM for 157PN (Fig. 3C) and 3.03 µM for AC17PN (Fig. 3D). Pharmacologic mTOR inhibition enhanced alpelisib’s suppressive effects on neurosphere growth in all GSC lines tested (Fig. 3E–H). However, the combination of alpelisib and OSI-027 reduced neurosphere growth more significantly in PN GSC lines, indicating this combination has more potent antineoplastic effects on this GBM subtype.

Given the potent inhibitory effects of combined PI3Kα and mTOR inhibition on GSC neurosphere growth, we next employed extreme limiting dilution analysis (ELDA)^51^ to estimate stem cell frequencies. Dual PI3Kα and mTOR inhibition potently disrupted glioma stem cell frequencies in all GSC lines tested (Fig. 3I–L). After combined PI3Kα and mTOR inhibition, stem cell frequencies decreased from 1 in 1 cell to 1 in 35.28 cells for 83Mes (Fig. 3M, upper panel), from 1 in 14.3 to 1 in 290.4 cells for JK16 (Fig. 3N, upper panel), from 1 in 1 to 1 in 18.91 cells for 157PN (Fig. 3O, upper panel), and from 1 in 2.49 to 1 in 359.89 for AC17PN cells (Fig. 3P, upper panel). The χ^2^ analysis revealed highly significant blockade of stem cell frequencies after dual PI3Kα and mTOR inhibition (Fig. 3M–P, lower panels). These results suggest a critical role for PI3Kα and mTOR in GSCs, and indicate that combined PI3Kα and mTOR inhibition may disrupt self-renewal capacities of GSCs.

### Increased expression and activity of the alpha catalytic PI3K isoform is associated with poor prognosis of the PN GBM subtype

**Figure 4:**
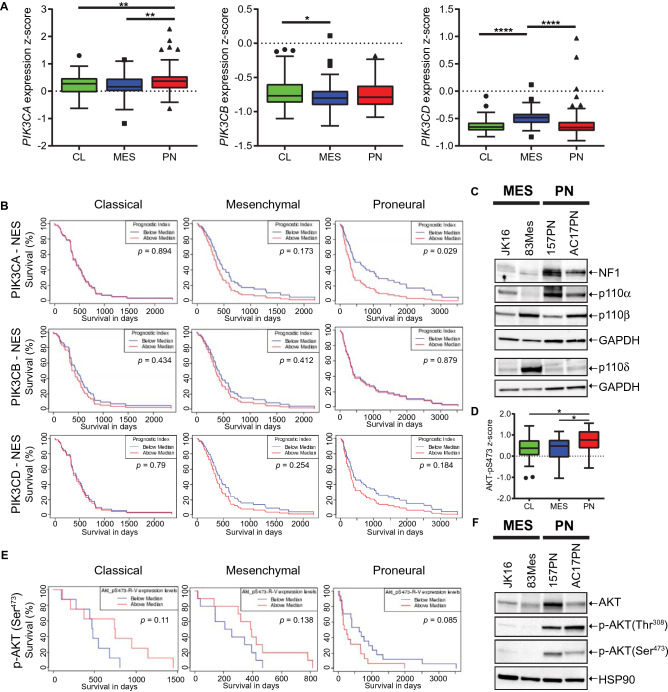
A discrete role for the alpha catalytic PI3K isoform in proneural (PN) GSCs. (**A**) Expression z-score data from TCGA (Extended Verhaak) for *PIK3CA* (left panel), *PIK3CB* (middle panel) and *PIK3CD* (right panel) were downloaded from the GBM-BioDP (http://gbm-biodp.nci.nih.gov/) for CL (n = 105), MES (n = 124) and PN (n = 113) subtype GBM from the HT Human Genome U133 (HT_HG-U133A) array and analysed using GraphPad Prism 8. As recent evidence suggests the neural subtype represents excessive contamination with normal brain, it has been excluded from the analysis. Unpaired one-way ANOVA, *p ≤ 0.05; **p ≤ 0.01; ****p ≤ 0.0001. (**B**) Survival analysis based on the impact of the multi-gene prognostic index for coexpression of *PIK3CA* and *NES* (upper panels), *PIK3CB* and *NES* (middle panels) or *PIK3CD* and *NES* (lower panels) for CL (n = 53, left panels), MES (n = 57, middle panels) and PN (n = 56, right panels) subtype. TCGA gene expression data (Verhaak Core) for *PIK3CA* and *NES*, *PIK3CB* and *NES* or *PIK3CD* and *NES* from the HT_HG-U133A array were used for multigene prognostic index. Figure was generated using the GBM-BioDP software. (**C**) GSC lines JK16, 83Mes, 157PN and AC17PN were analysed by Western blot using antibodies against NF1, p110α and GAPDH. Membrane was stripped and reprobed with an antibody against p110β. Lysates from the same experiment were run in parallel and probed for p110$\updelta$ and GAPDH. (**D**) Reverse-phase protein microarray (RPPA) data from TCGA GBM samples. Boxplots of RPPA analytes for phospho-AKT(Ser^473^) measured in CL (n = 28), MES (n = 29) and PN (n = 41) samples from the cohort of GBM tumours (Extended Verhaak) that was subjected to RPPA analysis within the TCGA consortium. RPPA data were downloaded from the GBM-BioDP and analysed using GraphPad Prism 8. Unpaired one-way ANOVA: *p ≤ 0.05. (**E**) Survival analysis based on RPPA analysis for phospho-AKT(Ser^473^) using the Extended Verhaak cohort submitted to the TCGA consortium for CL (n = 28, left panel), MES (n = 29, middle panel) and PN (n = 41, right panel) subtypes. Figure was generated using the GBM-BioDP software. (**F**) GSC lines JK16, 83Mes, 157PN and AC17PN were analysed by Western blot using antibodies against AKT. Membrane was stripped and re-probed for p-AKT(Thr^308^), then stripped and reprobed for p-AKT(Ser^473^), then stripped and re-probed for HSP90.

Recent efforts have identified three GBM-intrinsic transcriptional subtypes designated as proneural (PN), mesenchymal (MES), and classical (CL) GBM^5^. Using the GBM-BioDP portal (http://gbm-biodp.nci.nih.gov/)^52^ , we found that expression of *PIK3CA*, the gene encoding p110α, was significantly increased in the PN subtype (Fig. 4A, left panel) as compared to other class I_A_ catalytic PI3K isoforms (Fig. 4A). This elevated *PIK3CA* expression in PN suggests a unique role for PI3Kα in this subtype.

Evidence indicates important roles for PI3K/AKT signalling in GSCs^27,53^. We found that concurrent overexpression of *PIK3CA* with the neural stem cell marker *NES* was significantly associated with poor prognosis in the PN subtype, but not in the CL or MES subtypes (Fig. 4B, upper panels). Similarly, elevated coexpression of *PIK3CA* with additional pluripotency markers, such as *SOX2* (Supplementary Fig. S3A), *PROM1* (Supplementary Fig. S3B), *NANOG* (Supplementary Fig. S3C), or *CD44* (Supplementary Fig. S3D) exhibited an enhanced trend for poor prognosis in the PN subtype, and this trend was significant when *PIK3CA* was coexpressed with *PROM1* (*P* = 0.2; Supplementary Fig. S3B) or *CD44* (*P* = 0.04; Supplementary Fig. S3D). These data indicate that elevated coexpression of *PIK3CA* with pluripotency markers is associated with poor prognosis in PN GBM. Significantly, neither coexpression of *PIK3CB* with *NES* (Fig. 4B, middle panels) nor *PIK3CD* with *NES* (Fig. 4B, lower panels) served as a prognostic marker for survival in either GBM subtype. These data indicate elevated expression of p110α, but not p110β or p110$\updelta$ in PN GSCs might contribute to malignant tumour progression and worse prognosis in GBM.

Next we used our panel of GSC lines to assess expression of p110α in MES and PN GSCs. Expression of the tumour suppressor Neurofibromatosis Type 1 (NF1) was greatly reduced in JK16 and 83Mes GSC lines (Fig. 4C), corroborating they represent the mesenchymal subtype because hemizygous deletions of a region at 17q11.2, containing the NF1 gene, is a hallmark of this GBM subtype^4,5^. We observed that p110α expression was higher in PN GSC (157PN, AC17PN) lines as compared to their MES (83Mes, JK16) counterparts (Fig. 4C). To elucidate the downstream signalling effects of elevated p110α, we employed Reverse Phase Protein Array (RPPA) analysis using the GBM-BioDP portal. RPPA analysis revealed a significant increase in AKT(Ser^473^) phosphorylation in the PN subtype as compared to CL and MES patient samples (Fig. 4D). This suggests the PI3K/AKT pathway is activated in PN GBM, and this might stimulate GBM progression in this subtype. Indeed, we found a trend for phosphorylation of AKT on Ser-473 being associated with worse survival in PN, and this trend was inversed in the CL and MES subtypes (Fig. 4E). Additionally, we detected greatly increased phosphorylation of AKT on Ser-473 and Thr-308 in PN GSC neurospheres as compared to their mesenchymal counterparts (Fig. 4F). Together, these data indicate a discrete role for the alpha catalytic PI3K isoform and suggest that elevated p110α expression is associated with increased AKT activation in the GSC subpopulation and with worse prognosis in the PN GBM subtype.

## Discussion

Accumulating evidence suggests key roles for the PI3K/AKT/mTOR pathway in GBM, and this is underscored by the finding that roughly 90% of GBM exhibit at least one alteration in the RTK/PI3K/PTEN pathway^3^. Early reports indicated enhanced PI3K signalling is associated with poor prognosis in GBM^22^ and also described increased AKT signalling as one hallmark of poor outcome in the disease^54^. This raises the possibility of therapeutic targeting of PI3K for the treatment of GBM. As pan-PI3K inhibitors have limited potential due to a narrow therapeutic window^29^, we explored the effects of alpelisib, a PI3Kα selective inhibitor with a favourable safety profile that has shown promise in patients with a variety of solid tumours^32^. We found alpelisib inhibited phosphorylation of AKT(Ser^473^) and exhibited antineoplastic effects in GBM cell lines. These effects were enhanced when alpelisib was combined with pharmacological mTOR inhibition. This is in line with earlier studies, indicating mTOR activity can sustain survival after selective PI3Kα inhibition^34,55^. In order to study stem-like cancer cells in vitro, we propagated U87 and LN18 cell lines as spheroids in 3-D under stem cell conditions, as described before^56^. Resulting spheres exhibited substantially increased nestin expression, suggesting they adopted stem-like characteristics, consistent with previous reports^43,44,57^. Under these conditions, spheres exhibited greatly enhanced phosphorylation of AKT(Ser^473^), indicating increased PI3K/AKT pathway activation in this spheroid model. Importantly, this AKT phosphorylation on Ser-473 was efficiently blocked by alpelisib, indicating the alpha catalytic PI3K isoform is responsible for this increased AKT phosphorylation in GBM 3-D spheroids. This raises the possibility that stem-like cancer cells grown as 3-D spheroids are highly dependent on increased PI3K signalling for growth and survival. In line with this, alpelisib potently blocked spheroid growth and this effect was significantly enhanced when combined with pharmacological mTOR inhibition.

Expression of *PIK3CA* is highest in the PN subtype (see Fig. 4A). In addition, multi-gene prognostic index analysis revealed that poor prognosis is significantly associated with elevated coexpression of *PIK3CA* (but not *PIK3CB* or *PIK3CD*) with pluripotency markers *PROM1*, *CD44* and the neural stem/progenitor marker *NES* exclusively in the PN subtype. This suggests a discrete role for the alpha catalytic PI3K isoform in the GSC population of this subtype. In line with this, RPPA analysis revealed significantly elevated phosphorylation of AKT on Ser-473 in patients with the PN subtype, as compared to MES and CL subtypes, which is consistent with previous reports^3^. Concomitantly, there was a trend towards poor prognosis associated with p-AKT(Ser^473^) in PN, but not in MES or CL patient samples. Together, these data indicate a distinct dependency of PN GBM on PI3Kα signalling, which may result in greatly enhanced vulnerability of GSCs of the PN subtype to selective PI3Kα inhibition (Supplementary Fig. S4).

Recent studies have indicated GBMs contain hierarchies of MES and PN GSCs and their more differentiated progenitors, suggesting most cells fall within a mesenchymal-to-proneural axis, explaining why GSC lines representing the CL subtype have not yet been established^13,15^. We employed established GSC lines 83Mes, 157PN, AC17PN^19^ , and JK16^49,50^. In line with gene expression data (see Fig. 4A), we found p110α expression was highest in PN GSC lines, and phosphorylation of AKT on Ser-473 and Thr-308 was also greatly increased in PN as compared to MES GSC lines (see Fig. 4C,F). Taken together, gene expression data, RPPA analysis, and western blot analysis suggest PI3Kα pathway activation in GSCs of the PN subtype and raise the possibility of increased vulnerability of the PN subtype to selective PI3Kα inhibition. Indeed, while MES GSCs exhibited rather high IC_50_ values for inhibition of neurosphere growth, PN neurospheres were comparatively sensitive to alpelisib, and again these effects were significantly enhanced by simultaneous mTOR inhibition in PN GSC lines.

PI3K is a key mediator of platelet-derived growth factor receptor A (PDGFRA) signalling in GBM^58^ and alterations in *PDGFRA* constitutes a major feature of the PN GBM subtype^4^. Interestingly, in PN patient samples lacking *PDGFRA* abnormalities, *PIK3CA/PIK3R1* mutations were frequently observed^4^. Together, these findings indicate that activation of the PI3K/AKT pathway, either through increased PDGFRA signalling or aberrant activation of PI3Kα subunits, is a hallmark of PN GBM. In line with this, we found elevated expression and activation of PI3Kα in the PN subtype, which is associated with increased sensitivity to selective PI3Kα inhibition in PN GSCs.

CSCs have attracted increased interest because of their central roles in therapy resistance and tumour recurrence^59^ and, thus, targeting GSCs might be important for improving GBM clinical outcomes^45^. Besides characterizing PI3Kα as a promising target in PN GBM, we additionally provide further evidence for key roles of PI3Kα signalling in GSCs. There has already been some evidence for alterations in PI3K or PTEN signalling contributing to brain CSC function. For instance, previous studies using *Pten* conditional knockout mice demonstrated increased self-renewal ability of PTEN-deficient neural stem/progenitor cells^60,61^. Reciprocally, PI3K activation is required to promote self-renewal in embryonic stem cells^62^, and neural progenitors are prone to AKT-driven oncogenic transformation^25^. Additionally, increased PI3K/AKT activation in brain CSCs^27^ and activation of PI3K in CD133^+^ GSCs^28^ stimulate radioresistance. Together, these findings suggest key roles of PI3K signalling in GSCs that may promote self-renewal and therapy resistance. Here, we define this key role of PI3K signalling in GSCs is exceptionally important in GSCs of the PN subtype. PN GSCs depict greatly increased PI3Kα activation as judged by enhanced p110α expression and substantially increased phosphorylation of AKT. In line with this, PN GSC lines show increased sensitivity to specific PI3Kα inhibition, as compared to MES GSCs (Supplementary Fig. S4).

The antineoplastic effects of alpelisib can be enhanced by pharmacological mTOR inhibition in breast cancer^34,55^ and medulloblastoma^63^. Here we provide evidence that also in GSCs concomitant mTOR inhibition potentiates the antineoplastic effects of PI3Kα inhibition. This is in agreement with previous studies, suggesting that concurrent inhibition of PI3K and mTOR is required to efficiently reduce the self-renewal ability of neural stem/progenitor cells and to drive GSCs into differentiation^64,65^. Elucidating the molecular mechanisms that govern GSCs of a particular subtype might facilitate the development of novel targeted therapeutics for GBM. Our study provides valuable insights into subtype selective PI3Kα dependencies in GBM and GSCs. Specifically, we highlight a previously underappreciated dependency of the PN subtype on increased PI3Kα signalling that may translate into enhanced vulnerability of PN GBM to PI3Kα inhibition. The enhanced effects observed in GBM tumour cells and GSCs support the necessity of dual PI3Kα and mTOR inhibition to efficiently disrupt GSCs (Supplementary Fig. S4)^64,66^. Future studies to better understand the subtype selective PI3Kα dependencies in GSCs, as well as additional preclinical studies using dual PI3Kα/mTOR inhibition in orthotopic patient-derived xenograft (PDX) models may have important clinical-translational implications for the treatment of GBM.

## Materials and methods

### Cell culture and reagents

For conventional 2-D adherent culture, U87 (*PTEN* mutated), LN18 (*PTEN* wild-type) and LN443 (*PTEN* mutated) GBM cells were grown in DMEM supplemented with 10% FBS (Thermo Fisher) and gentamycin (1 mg/ml). U87 and LN18 3-D spheroid cultures were propagated in cancer stem cell (CSC) medium consisting of DMEM/F12 supplemented with B27 (2%), EGF (20 ng/ml), bFGF (20 ng/ml), heparin (5 µg/ml) and gentamycin (1 mg/ml), as described previously^56,67^. Glioma stem cell (GSC) lines 83Mes, 157PN and AC17PN were subtyped and described before^19^. Also, the JK16 GSC line was described previously^50^. GSC lines were propagated as 3-D neurospheres in CSC medium. All cell lines were regularly tested for mycoplasma contamination and subjected to short-tandem repeat (STR) analysis (Genetica DNA Laboratories) to ensure genetic stability. U87, LN18 and LN443 were authenticated using published reference STR profiles. The most recent STR analysis was done in March/April 2020. The PI3Kα specific inhibitor alpelisib (BYL719) and the mTOR inhibitor OSI-027 were purchased from ChemieTek and both were dissolved in DMSO as a vehicle.

### Cell viability, soft agar assays and confocal laserscanning microscopy

To determine cell viability, the WST-1 assay kit (Roche) was used as described^68^. The CytoSelect 96-Well Cell Transformation Assay (Cell Biolabs) was used to determine anchorage-independent growth as described^68^. For confocal laserscanning microscopy, U87 and LN18 cells and spheroids were processed and imaged as described before^69^.

### Neurosphere assay and extreme limiting dilution analysis (ELDA)

For neurosphere assays, cells were seeded into Ultra-Low Attachment Round Bottom 96-Well Plates (Thermo Fisher/Corning) at 500 cells per well and processed as described previously^56,69^. ELDA was done as described before^56,69^ with the exception that GSCs were allowed to grow for 12 days. The Cytation 3 software (Gen5 v 2.09) was used to measure neurosphere diameters and only neurospheres with a diameter of ≥ 300 µm (83Mes), ≥ 50 µm (JK16), ≥ 75 µm (157PN), ≥ 75 µm (AC17PN), were scored positive for ELDA analysis (http://bioinf.wehi.edu.au/software/elda/)^51^.

### Bioinformatics and statistical analysis

*PIK3CA*, *PIK3CB* and *PIK3CD* gene expression data were downloaded from the GBM Bio Discovery Portal (GBM-BioDP) (http://gbm-biodp.nci.nih.gov/) using the Extended Verhaak dataset^4,52^ and analysed using GraphPad Prism 8. Similarly, protein Reverse Phase Protein Microarray (RPPA) data of the Extended Verhaak dataset^4^ for p-AKT^(S473)^ were downloaded and analysed using GraphPad Prism 8. Survival analysis of TCGA gene expression data for patients from the Verhaak Core^4^ was performed using the multigene prognostic index from the GBM-BioDP. For survival analysis interrogating the phosphorylation status of AKT on Ser-473, RPPA data from the Extended Verhaak dataset^4^ were analysed using the GBM-BioDP software. GraphPad Prism 8 was used for statistical analysis including calculating IC_50_ values. One-way analysis of variance (ANOVA) was used to compare more than two groups followed by Tukey test.

### Cell lysis and immunoblotting

Cells or neurospheres were treated with alpelisib or OSI-027 at the indicated concentrations for 90 min. Cells or neurospheres were lysed in phosphorylation lysis buffer (50 mM HEPES, 150 mM NaCl, 1 mM MgCl_2_, 0.5% Triton, 10% glycerol, 0.5% sodium deoxycholate, pH 7.9) supplemented freshly with 1 mM 1,4-Dithiothreitol (DTT), phosphatase and protease inhibitors (Roche). Cell extracts were processed as described before^69^. Antibodies against phospho-AKT(Ser^473^), phospho-AKT(Thr^308^), AKT, p110α, and p110β were purchased from Cell Signalling Technology. Antibody for p110δ was from Abcam. Antibodies against GAPDH were purchased from Millipore. Antibodies against HSP90 and NF1 were purchased from Santa Cruz. Following incubation with primary antibodies, membranes were incubated with anti-mouse horseradish peroxidase (HRP)-conjugated antibody (BioRad), or anti-rabbit HRP-conjugated antibody (GE Healthcare) and anti-mouse-AF488 antibody (Thermo Fisher) simultaneously, and visualized in a ChemiDoc MP Imaging System (BioRad). Uncropped blots as well as a comprehensive list of antibodies used in this study can be found in Supplementary Information 2.

## Supplementary information

Supplementary Figures.

Supplementary Information.
