# Supplementary material for: Combined PI3Kα-mTOR Targeting of Glioma Stem Cells
Source: Sci Rep. 2020 Dec 14;10:21873. doi: 10.1038/s41598-020-78788-z (PMC7736588; doi:10.1038/s41598-020-78788-z)
Supplement: Supplementary file 1 — Supplementary Figures. [file 41598_2020_78788_MOESM1_ESM.docx]

**Title:** Combined PI3Kα-mTOR Targeting of Glioma Stem Cells

**Authors:** Frank Eckerdt^1,2^, Jonathan B. Bell^1^, Christopher Gonzalez^1^, Michael S. Oh^1^, Ricardo E. Perez^1,3^, Candice Mazewski^1,3^, Mariafausta Fischietti^1,3^, Stewart Goldman^1,4^, Ichiro Nakano^5^ and Leonidas C. Platanias^1,3,6.^

**Institutions:**

^1^Robert H. Lurie Comprehensive Cancer Center of Northwestern University, Chicago, IL, USA

^2^Department of Neurological Surgery, Feinberg School of Medicine, Northwestern University, Chicago, IL, USA

^3^Division of Hematology/Oncology, Department of Medicine, Feinberg School of Medicine, Northwestern University, Chicago, IL, USA

^4^Division of Hematology/Oncology/Stem Cell Transplantation, Department of Pediatrics, Ann & Robert H. Lurie Children’s Hospital of Chicago, Chicago, IL, USA

^5^Department of Neurosurgery and O’Neil Comprehensive Cancer Center, University of Alabama at Birmingham, Birmingham, AL, USA

^6^Medicine Service, Jesse Brown VA Medical Center, Chicago, IL, USA


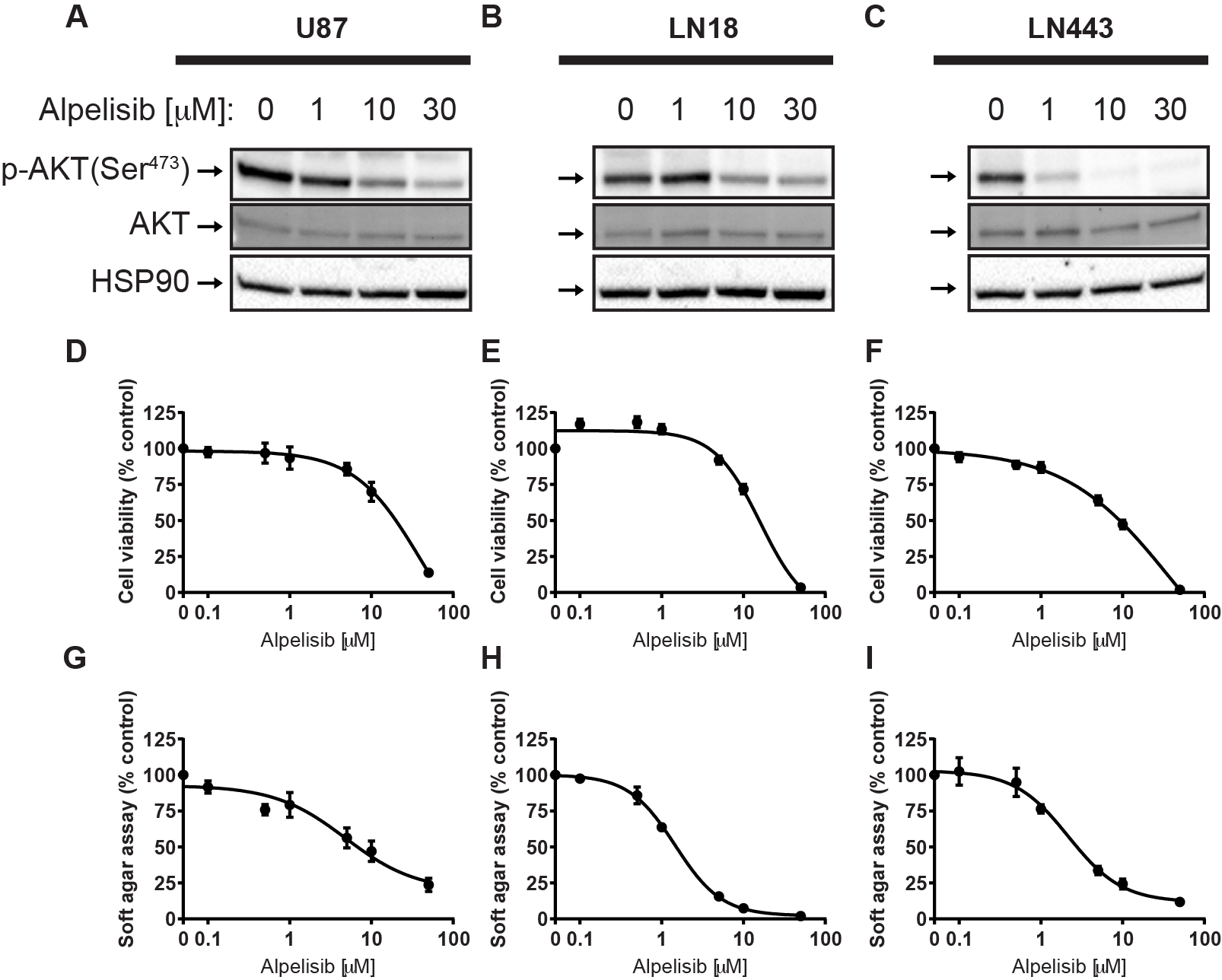


**Supplementary Figure S1:** Alpelisib inhibits PI3K/AKT signalling and exhibits antineoplastic effects in GBM cells. (A-C) U87 (A), LN18 (B) or LN443 (C) GBM cells were treated with increasing concentrations of alpelisib for 90 minutes and subjected to immunoblotting using mouse anti AKT and rabbit anti p-AKT(Ser^473^) antibodies simultaneously, followed by detection using anti-rabbit HRP and anti-mouse AlexaFlour488 secondary antibodies. Membranes were stripped and reprobed for mouse anti HSP90 antibody followed by detection using anti-mouse HRP antibody. (D-F) U87 (D), LN18 (E) or LN443 (F) GBM cells in 96-well plates (2,000 cells per well) were treated with increasing concentrations of alpelisib as indicated for 5 days and cell viability was determined using the cell proliferation reagent, WST-1. Data represent means ± SEM of 4 (U87 and LN18) or 3 (LN443) independent experiments, each done in triplicate. (G-I) U87 (G), LN18 (H) or LN443 (I) cells were seeded in soft agar in 96-well plates (2,500 cells per well) and treated with increasing concentrations of alpelisib, as indicated. After 7 days, colony formation was quantified using the fluorescent CyQUANT GR Dye. Data represent means ± SEM of 4 independent experiments, each done in triplicate.


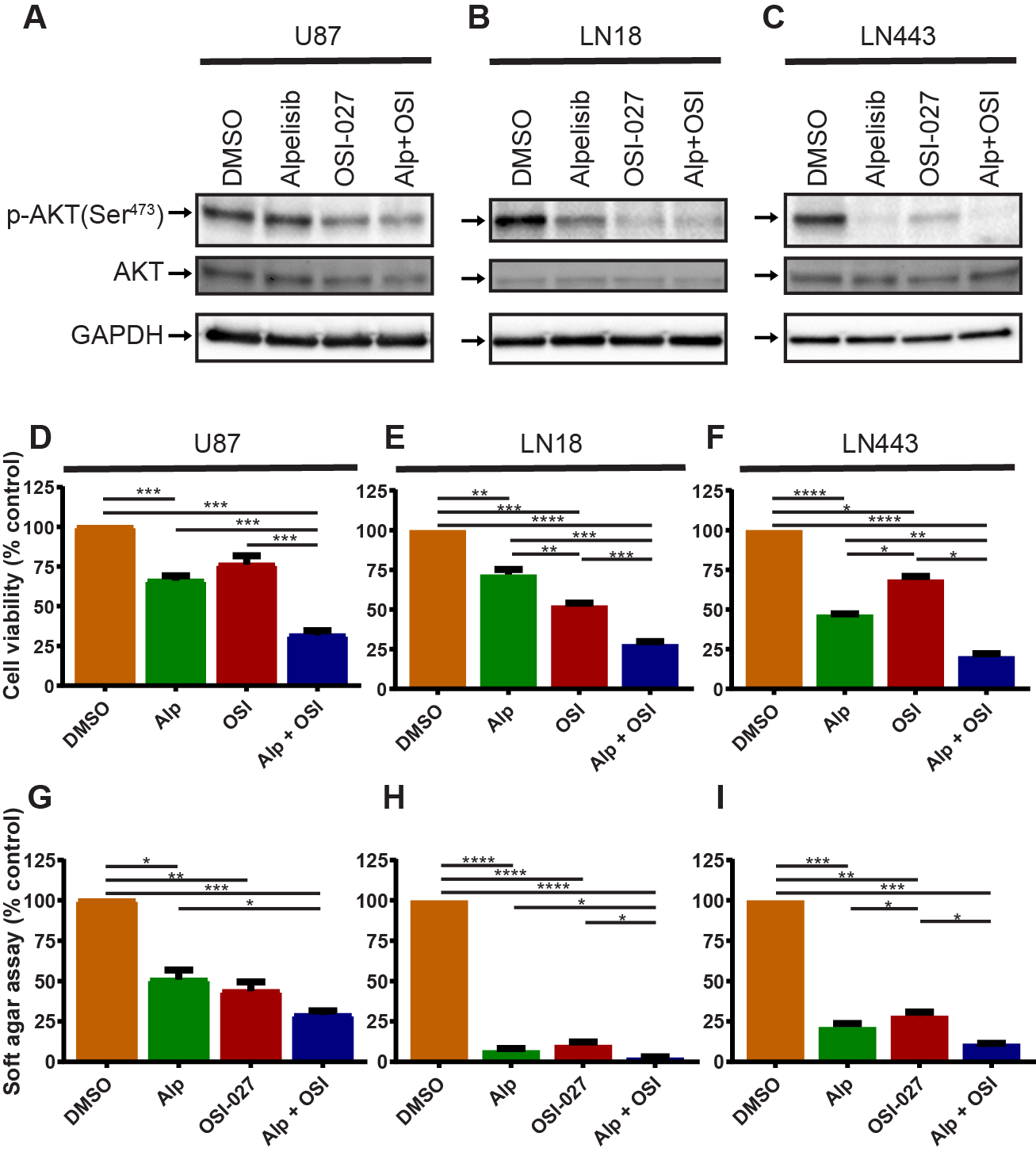


**Supplementary Figure S2:** The catalytic mTOR inhibitor OSI-027 enhances the inhibitory effects of alpelisib on GBM cells. (A-C) U87 (A), LN18 (B) and LN443 (C) cells were treated with alpelisib (10 µM) and/or OSI-027 (5 µM) for 90 minutes and subjected to immunoblotting using mouse anti AKT and rabbit anti p-AKT(Ser^473^) antibodies simultaneously, followed by detection using anti-rabbit HRP and anti-mouse AlexaFlour488 secondary antibodies. Lysates were run in parallel and immunoblotted with antibodies against GAPDH. (D-F) U87 (D), LN18 (E) and LN443 (F) cells were seeded into 96-well plates (2,000 cells per well) and incubated with alpelisib (10 µM) and/or OSI-027 (2 µM). After 5 days, cell viability was quantified using the cell proliferation reagent, WST-1. Data represent means ± SEM of 4 (U87, LN18) or 3 (LN443) independent experiments, each done in triplicate. Unpaired one-way ANOVA, *, *p* ≤ 0.05; **, *p* ≤ 0.01; ***, *p* ≤ 0.001; ****, *p* ≤ 0.0001. (G-I) U87 (G), LN18 (H) and LN443 (I) cells were seeded into 96-well plates in soft agar (2,500 cells per well) and incubated with alpelisib (10 µM) and/or OSI-027 (2 µM). After 7 days, colony formation was quantified using the fluorescent CyQUANT GR Dye. Data represent means ± SEM of 4 (U87, LN18) or 3 (LN443) independent experiments, each done in triplicate. Unpaired one-way ANOVA, *, *p* ≤ 0.05; **, *p* ≤ 0.01; ***, *p* ≤ 0.001; ****, *p* ≤ 0.0001.


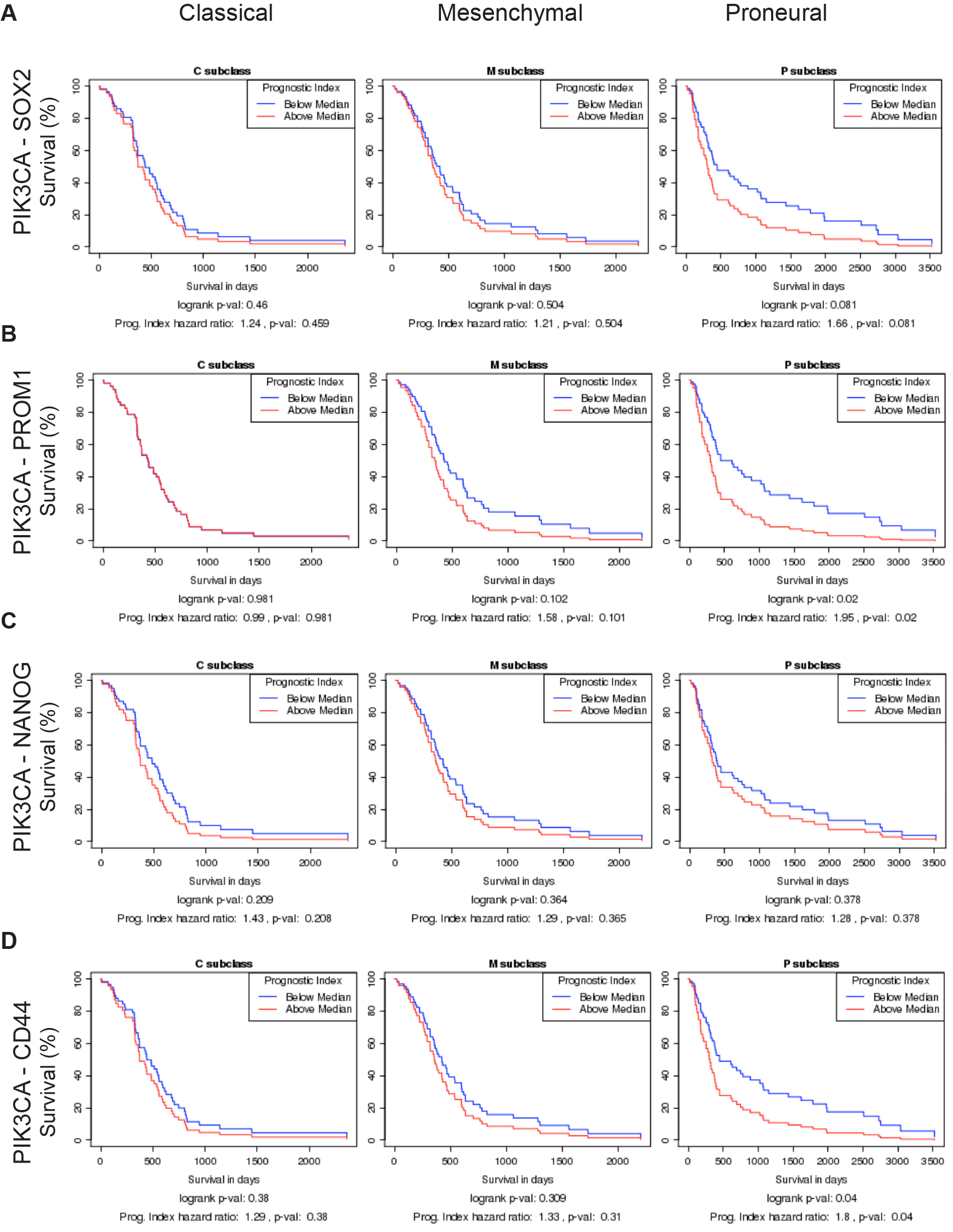


**Supplementary Figure S3:** Prognostic significance for PIK3CA coexpression with pluripotency markers in GBM subtypes. Survival analysis based on the impact of the multi-gene prognostic index for coexpression of *PIK3CA* and *SOX2* (A), *PROM1* (B), *NANOG* (C), or *CD44* (D) for CL (left panels), MES (middle panels) and PN (right panels) subtypes. TCGA gene expression data (Verhaak Core) from the HT_HG-U133A array were used for multigene prognostic index. Figure was generated using the GBM-BioDP software.


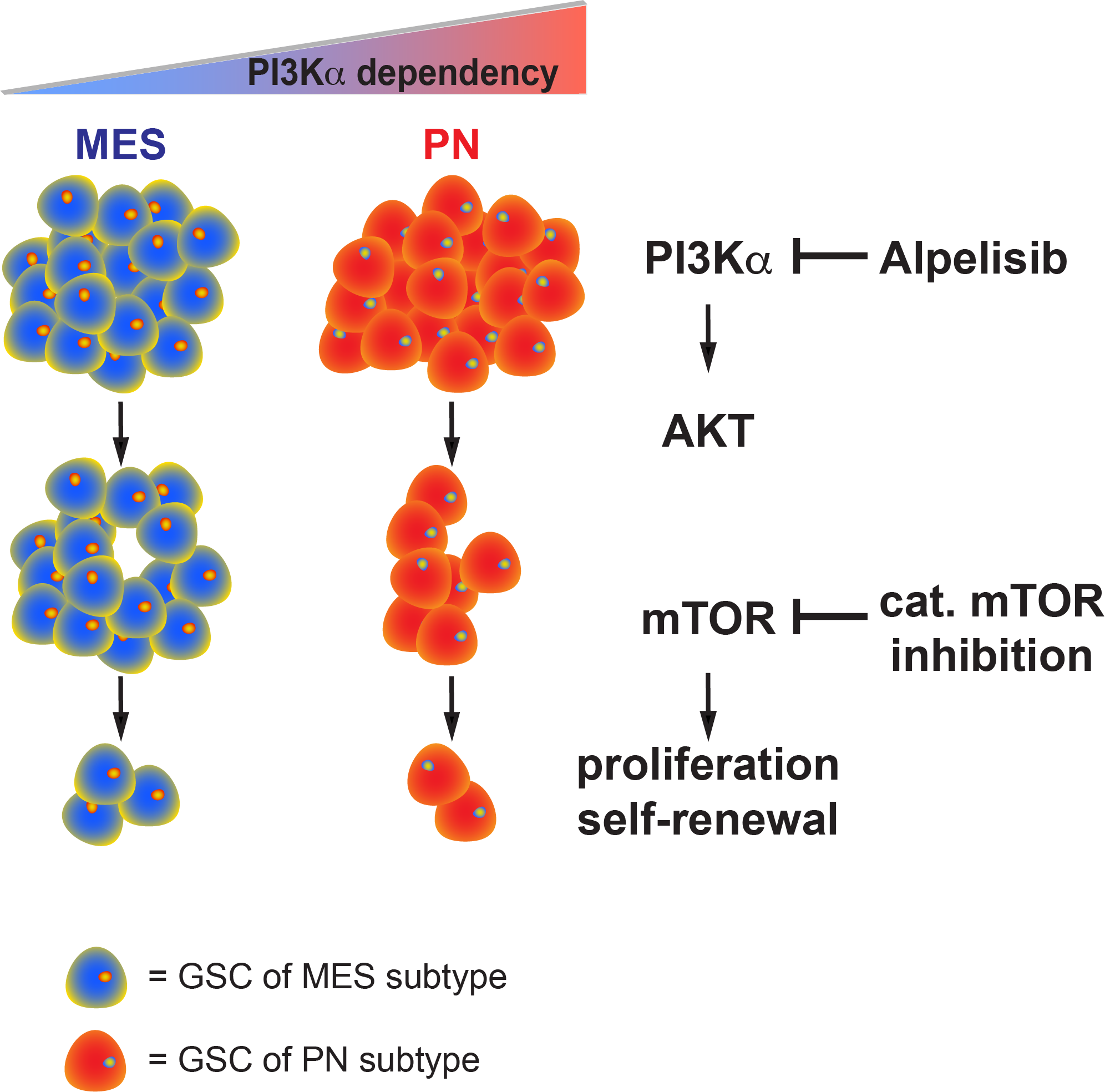


**Supplementary Figure S4:** Proposed model of MES and PN responses to combined PI3Kα and mTOR inhibition. Treatment with a catalytic PI3Kα inhibitor (alpelisib), blocks growth of PN (red) more potently than MES (blue) GSCs due to increased dependency of PN GSCs to PI3Kα signaling. Combining a catalytic mTOR inhibitor with the catalytic PI3Kα inhibitor further blocks growth and disrupts cancer stem cell frequencies in both PN and MES GBM. Figure was generate using Adobe Illustrator 2019, v23.1.1 for Mac.
