# Supplementary material for: Combined PI3Kα-mTOR Targeting of Glioma Stem Cells
Source: Sci Rep. 2020 Dec 14;10:21873. doi: 10.1038/s41598-020-78788-z (PMC7736588; doi:10.1038/s41598-020-78788-z)

Combined PI3Kα-mTOR Targeting of Glioma Stem Cells

Frank Eckerdt[1,2], Jonathan B. Bell[1], Christopher Gonzalez[1], Michael S. Oh[1], Ricardo E. Perez[1,3], Candice Mazewski[1,3], Mariafausta Fischietti[1,3], Stewart Goldman[1,4], Ichiro Nakano[5] and Leonidas C. Platanias[1,3,6].

[1]Robert H. Lurie Comprehensive Cancer Center of Northwestern University, Chicago, IL, USA

[2]Department of Neurological Surgery, Feinberg School of Medicine, Northwestern University, Chicago, IL, USA

[3]Division of Hematology/Oncology, Department of Medicine, Feinberg School of Medicine, Northwestern University, Chicago, IL, USA

[4]Division of Hematology/Oncology/Stem Cell Transplantation, Department of Pediatrics, Ann & Robert H. Lurie Children's Hospital of Chicago, Chicago, IL, USA

[5]Department of Neurosurgery and O'Neil Comprehensive Cancer Center, University of Alabama at Birmingham, Birmingham, AL, USA

[6]Medicine Service, Jesse Brown VA Medical Center, Chicago, IL, USA

Running Title: Dual PI3Kα and mTOR Inhibition in GBM

Keywords: Glioblastoma, glioma stem cells, PI3K, mTOR, alpelisib

Correspondence to: Frank D. Eckerdt, Robert H. Lurie Comprehensive Cancer Center, 303 East Superior Street, Lurie 3-220, Chicago, IL 60611, Tel. 312-503-0292; Email: frank.eckerdt@northwestern.edu

Supplemental Table 1

ANTIBODY	SOURCE	IDENTIFIER
Pan AKT (40D4)	Cell Signaling Technology	Cat# 2920 RRID:AB_1147620
p-AKT(Ser^{473}) (D9E)	Cell Signaling Technology	Cat# 4060 RRID:AB_2315049
p-AKT(Thr^{308}) (C31E5E)	Cell Signaling Technology	Cat#:2965 RRID:AB_2255933
HSP-90 (H-114)	Santa Cruz Biotechnology	Cat#:sc-7947 RRID:AB_2121235
GAPDH	Millipore	Cat# MAB374, RRID:AB_2107445
Nestin (10C2)	StemCell Technologies	Cat#:60091 RRID:AB_2650581
PI3 Kinase p110α (C73F8)	Cell Signaling Technology	Cat# 4249 RRID: AB_2165248
PI3 Kinase p110β (C33D4)	Cell Signaling Technology	Cat# 3011 RRID:AB_2165246
PI3 Kinase p110δ	Abcam	Cat# ab1678 RRID: 302513
Neurofibromin	Santa Cruz Biotechnology	Cat# sc-67 RRID:AB_21496819
Donkey anti-Mouse IgG (H+L) Alexa Fluor 488	ThermoFisher Scientific	Cat# A21202 RRID:AB_141607
Rabbit IgG HRP Linked Whole Ab	GE Healthcare	NA934-1ML RRID:AB_772206
Goat Anti-Mouse IgG (H + L)-HRP Conjugate	Biorad	Cat#:1706516 RRID:AB_11125547

Supp Table 1: List of antibodies used in this study, their manufacturers, catalog numbers and Research Resource Identifier (RRID) numbers.

Related to Supplementary Figure S1 full blots (Biorad ChemiDoc)

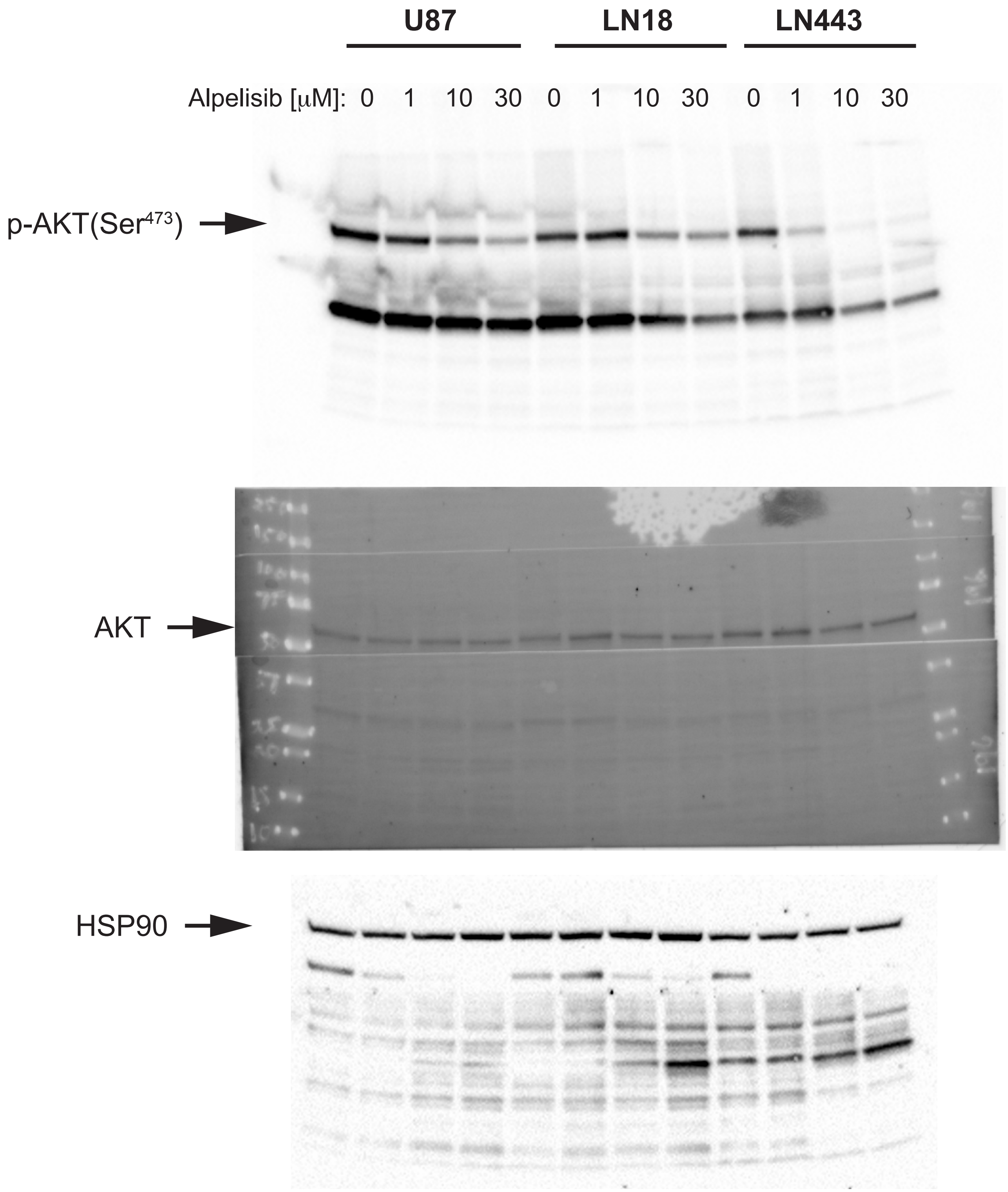

Related to Supplementary Figure S2 full blots (Biorad ChemiDoc)

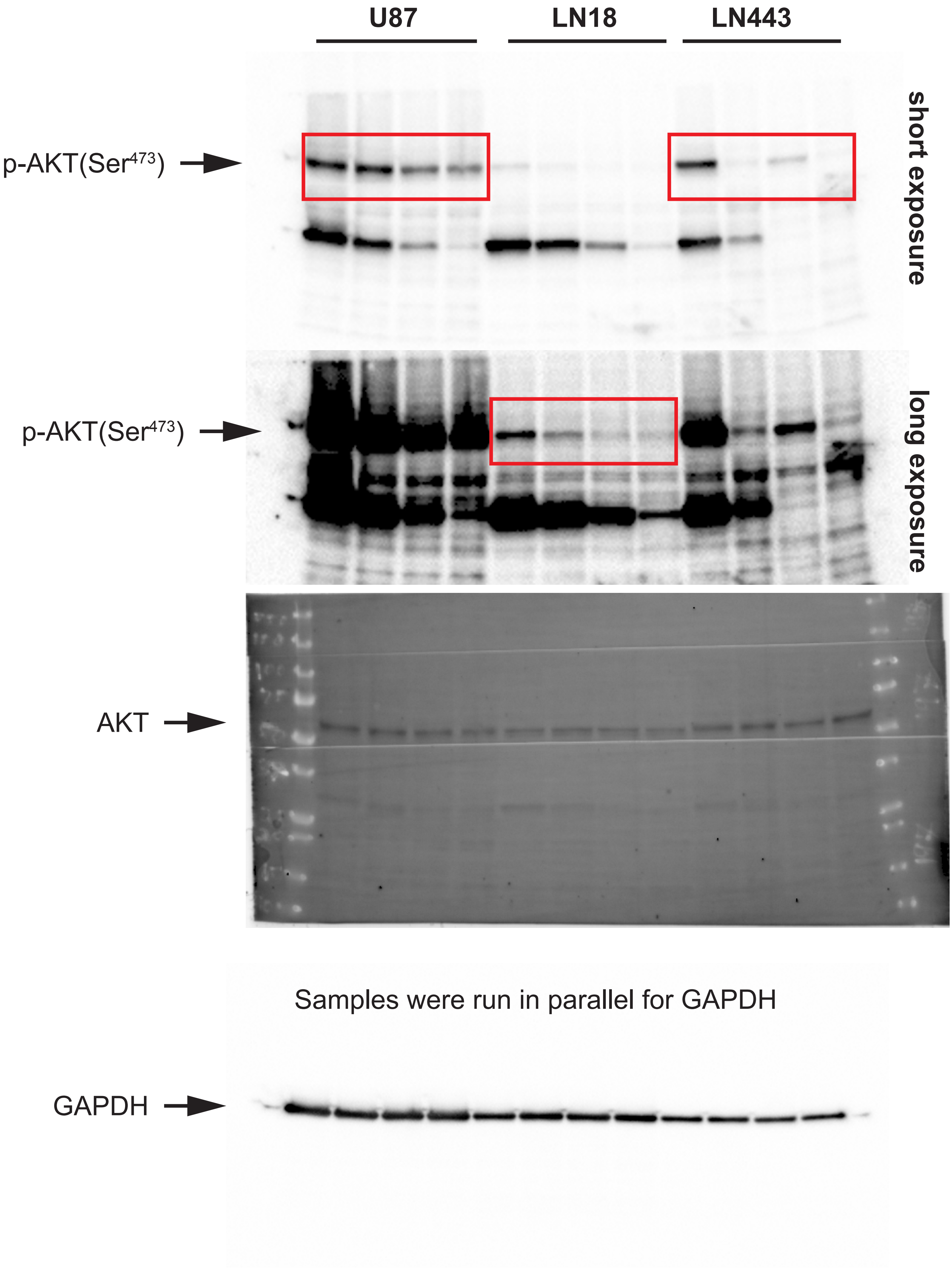

Related to Figure 1 full blots (Biorad ChemiDoc)

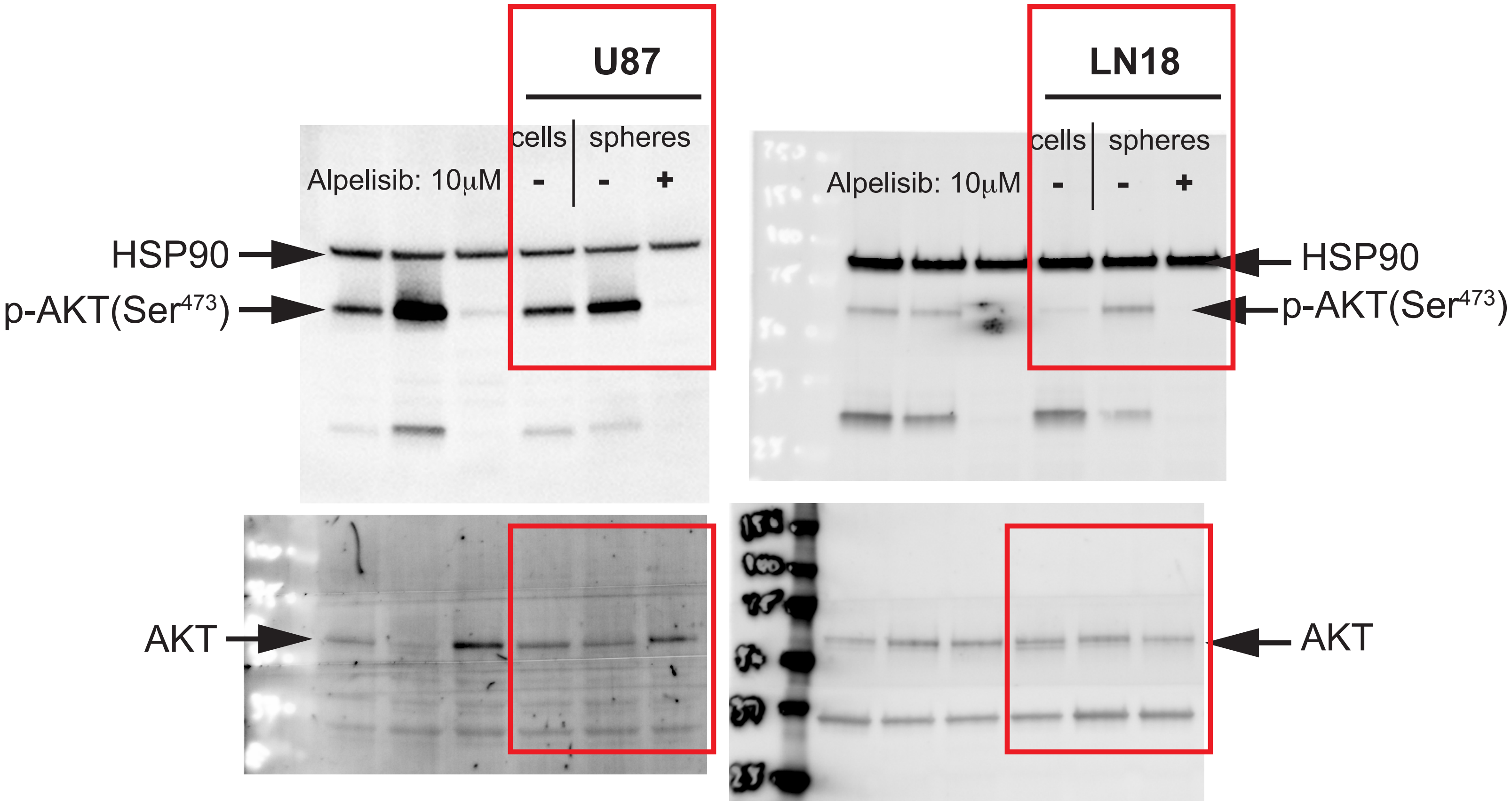

Related to Figure 4 full blots (Biorad ChemiDoc) (4C, red; 4F, green)

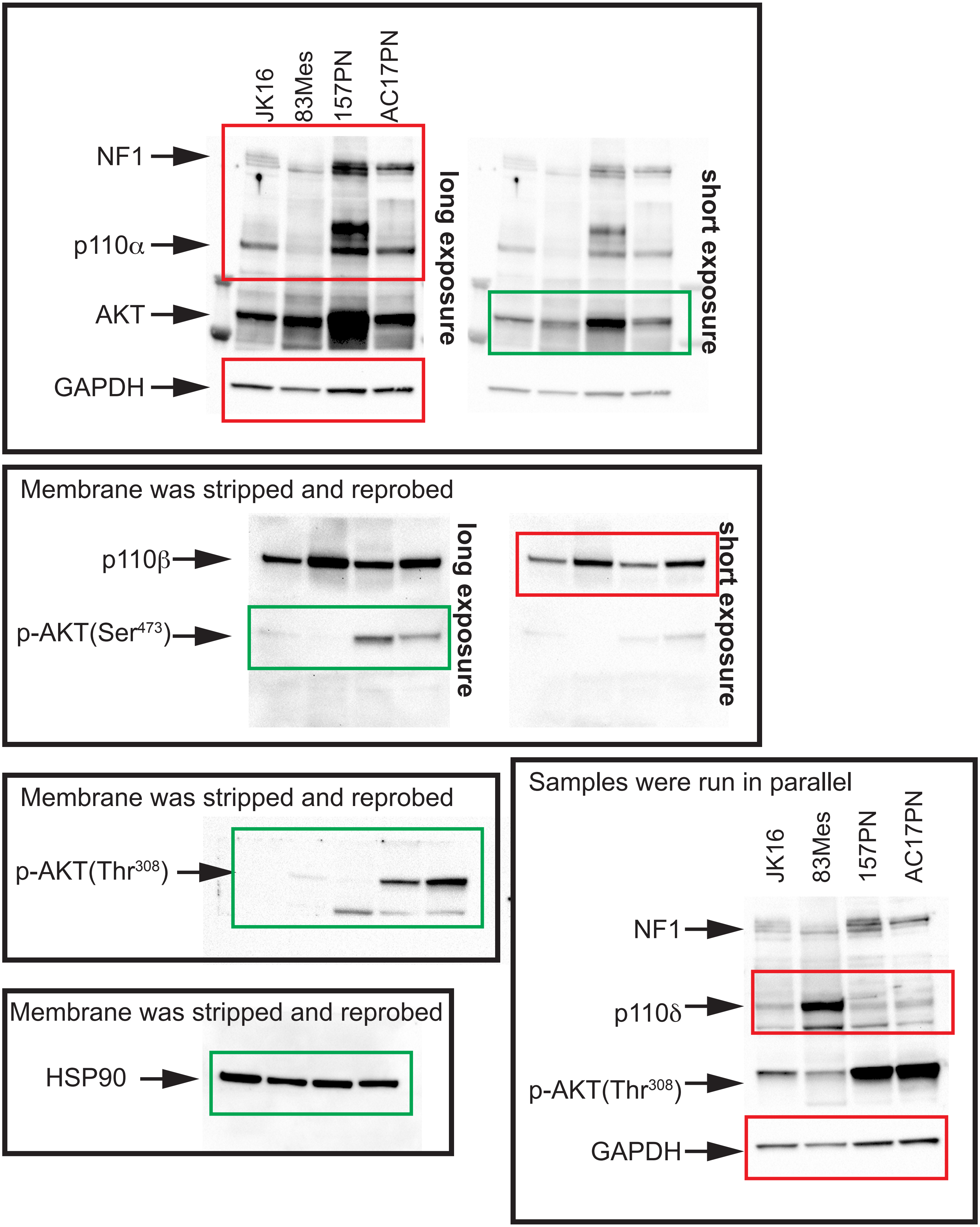

Supplement: Supplementary file 2 — Supplementary Information. [file 41598_2020_78788_MOESM2_ESM.pdf]
